# Dr. Samuel Bruce Craton

**Published:** 1915-04

**Authors:** 


**Dr. Samuel Bruce Craton,** Syracuse, 1890; died at his home in Syracuse, February 26, of pneumonia, aged about 50. He was educated at Wolford College, Spartenburg, South Carolina, studied pharmacy for a year and then entered the Syracuse College of Medicine, graduating in 1890. After practicing a year with Dr. U. H. Brown, an oculist of Syracuse, he went to New York for post graduate study and opened an office in the University Building which he occupied till his death.

